# 2128. Lack of Congenital Transmission in Infants Born of Female Patients with Chagas Disease who Became Pregnant During a Nifurtimox Study (CHICO and CHICO SECURE Study)

**DOI:** 10.1093/ofid/ofac492.1749

**Published:** 2022-12-15

**Authors:** A L T C H E H Jaime, Teresa Ramirez, Victor Sierra, Guillermo Moscatelli, Juan Dib, Jimy Pinto, Lourdes Ortiz, Andrea Falaschi

**Affiliations:** Hospital de Niños R. Gutierrez, Buenos Aires, Ciudad Autonoma de Buenos Aires, Argentina; Centro de Chagas, Hospital Independencia, Santiago del Estero, Santiago del Estero, Argentina; CAIMED, PI, Bogota, Distrito Capital de Bogota, Colombia; Hospital de Niños R. Gutierrez, Buenos Aires, Ciudad Autonoma de Buenos Aires, Argentina; Fundación Salud Para el Trópico, Santa Marta, Casanare, Colombia; CEADES, Cochabamba, Cochabamba, Cochabamba, Bolivia; CEADES, Tarija, Tarija, Bolivia; Epidemiologia, HospitalDr H Notti, Mendoza, Mendoza, Argentina

## Abstract

**Background:**

There is some concern about the safety of nifurtimox (NF) for the offspring of treated women. Potential genotoxicity of NF was reported in in vitro models. However, the evidence to support the risks in fetal development is lacking for humans. In addition, a preventative effect over transplacental transmission was reported in girls and women with *T.cruzi* infection treated before pregnancy (mainly with benznidazole).

**Methods:**

A multicenter, randomized, double-blind phase 3 clinical trial (NCT02625974) was set up to evaluate safety and efficacy of NF. A total of 330 patients, younger 18 years old, were enrolled, 67 were females of child-bearing age.

The enrolled females of childbearing age agreed to use an effective method of contraception until 3 months after the last administration of NF. Pregnancy tests were performed at screening, during treatment and 30 days after end of treatment.

**Results:**

No pregnancies were reported during treatment period. There were 5 pregnancies reported during the 1^st^ year of post-treatment follow-up and another 21 pregnancies during the additional 3-year follow-up period. Two mothers did not consent to have their babies tested for *T cruzi* infection, and in one case an abortion was reported. Two women gave birth twice. There was information about transplacental transmission available in 18 babies. All mothers were *T.cruzi* RT-PCR negative at the end of the treatment period and remained negative during follow up.

All newborns were live, healthy full-term and of weight appropriate for their gestational age, and had no perinatal complications except one baby who was healthy but small for gestational age. *T.cruzi* infection was ruled out by a negative direct parasitological test during the first days of life in 12 infants , and /or by ELISA *T.cruzi* serology at 8 -12 months of age in 14 infants, which was negative in all cases.

**Conclusion:**

Our results showed that treatment of females on childbearing age with nifurtimox may prevent transplacental transmission of CD as was reported previously for benznidazole.

**Disclosures:**

**Jaime ALTCHEH, Sr., MD,PhD**, Bayer: Advisor/Consultant.

